# Vaccination against human papillomavirus in Brazilian schoolchildren: National Survey of School Health, 2019

**DOI:** 10.1590/1518-8345.6296.3834

**Published:** 2022-11-28

**Authors:** Isabella de Alcântara Gomes Silva, Ana Carolina Micheletti Gomide Nogueira de Sá, Elton Junio Sady Prates, Deborah Carvalho Malta, Fernanda Penido Matozinhos, Tércia Moreira Ribeiro da Silva

**Affiliations:** 1 Universidade Federal de Minas Gerais, Escola de Enfermagem, Departamento Materno Infantil e Saúde Pública, Belo Horizonte, MG, Brazil; 2 Scholarship holder at the Conselho Nacional de Desenvolvimento Científico e Tecnológico (CNPq), Brazil

**Keywords:** Papillomaviridae, Adolescent Health, Immunization, Papillomavirus Vaccines, Vaccination Refusal, Nurses

## Abstract

**Objective:**

to analyze the prevalence of schoolchildren vaccinated against human papillomavirus (HPV) and the reasons related to non-vaccination.

**Method:**

cross-sectional study, with data from the 2019 National Survey of School Health. The sample consisted of 160,721 students aged 13 to 17 years. The prevalence and confidence intervals (95%CI) of vaccinated adolescents were estimated according to location, sex, and administrative dependence of the school. The differences between the strata were evaluated with the Chi-square test. Adjusted prevalence ratios (aPR) and 95%CI were estimated with the Poisson regression model.

**Results:**

most of the students were vaccinated (62.9%), and the prevalence of girls (76.1%) was higher than that of boys (49.1%). The most prevalent reason for not vaccinating was “did not know they had to take” (46.8%), with the highest aPR in public schoolchildren in Brazil (1.6; 95%CI 1.5;1.7), from the Northeast region (1.2; 95%CI 1.1;1.2), and in students from private schools in the Northeast regions (1.1; 95%CI 1.1;1.2) and North (1.3; 95%CI 1.2;1.4).

**Conclusion:**

one out of every two Brazilian schoolchildren was vaccinated against HPV. Misinformation was a recurring reason for non-vaccination. The North and Northeast regions had the highest prevalence of non-vaccinated people, observed mainly in adolescents from public schools.

## Introduction

The National Program for Immunization (*Programa Nacional de Imunizações* - PNI), established in 1973 by the Unified Health System (SUS), is responsible for coordinating immunization actions and offering free immunobiologicals in Brazil^1^. In 2004, the PNI established a timetable for the adolescent public, which included the following vaccines: hepatitis B, double bacterial with the tetanus and diphtheric components (dT), the human papillomavirus vaccine (HPV), and the quadrivalent meningococcal ACWY vaccine^1^.

The HPV vaccine prevents lesions in the female and male genital organs and persistent infections caused by subtypes 6, 11, 16, and 18 of human papillomavirus, with types 16 and 18 considered oncogenic and potentially precursors of cervical cancer. In 2019, 5,880,000 new cases of cervical cancer were reported worldwide^2^ ^), (^ ^3^. In Brazil, in 2018 and 2019, approximately 16,370 new cases of cervical cancer were detected, occupying the third place in incidence among malignant tumors^4^. For each year of the triennium 2020-2022, 16,590 new cases of the disease are expected to occur^5^.

HPV vaccination prior to the onset of sexual activity^6^ ^), (^ ^7^ ^)^ is one of the pillars of the global strategy to eliminate cervical cancer, published by the World Health Organization (WHO) in 2020^8^. In Brazil, HPV vaccination began in 2014^9^. However, within the same period, the goal of immunizing 80% of girls aged between 9 and 14 years and boys aged 11 to 14 years was not reached in any of the federative units^9^.

Lack of knowledge about HPV and HPV vaccine, mistrust of vaccine safety and efficacy, lack of time, fear of pain, and negative experiences with vaccination are factors that impair the acceptance of the HPV vaccine by the adolescent public and are frequently listed by international studies^10^ ^), (^ ^11^. These factors, in addition to offering barriers to vaccination of adolescents against HPV^11^, compromise the achievement of vaccination coverage goals and increase the number of individuals susceptible to HPV, representing a public health problem^10^. However, studies investigating the barriers to vaccination of Brazilian adolescents against HPV are still very limited.

The particularities of adolescence - a phase marked by biopsychosocial transformations - and the need to understand the risk and protective factors regarding the health of this population motivated the launch of the first national survey aimed at the adolescent public in Brazil. In its fourth edition, the National Survey of School Health (PeNSE) investigated, among several aspects, the HPV vaccination situation and the reasons why adolescents aged 13 to 17 years, enrolled in public and private schools, were not vaccinated^12^.

Considering that in Brazil immunization actions are mostly coordinated by nurses^13^, understanding the reasons why adolescents are not vaccinating against HPV may support the adoption of culturally appropriate, flexible strategies that make adolescents and their guardians aware of the importance of HPV vaccination^14^. Furthermore, investigating these reasons may raise public health policies aimed at improving immunization indicators, a goal included in the United Nations 2030 Agenda for sustainable development goals^15^.

This is the first study that investigated the reasons for non-vaccination against HPV, using the PeNSE 2019 database. Considering the importance of understanding why adolescents are not being vaccinated, this study aims to analyze the prevalence of Brazilian adolescents vaccinated against HPV and the reasons pointed out by them for not to being vaccinated, according to PeNSE data, 2019 edition.

## Method

### Study design

Cross-sectional study, with data from the 2019 edition of PeNSE^12^. The research investigated the prevalence and distribution of risk and health protection factors of students enrolled in and who regularly attended the 7^th^ to 9^th^ grade of primary education (former 6^th^ to 8^th^ grade) and the 1^st^ to 3^rd^ grades of secondary education (morning, afternoon, and evening shifts), in public and private schools in Brazil. In this study, in order to ensure the presentation of essential information, the recommendations from Strengthening the Reporting of Observational Studies in Epidemiology (STROBE)^16^ were adopted.

### Context

PeNSE was conducted by the Brazilian Institute of Geography and Statistics (IBGE) in partnership with the Ministry of Health, from April 8 to September 30, 2019. In the questionnaire answered by the schoolchildren, the questions were organized into 14 thematic blocks, and the questions from the “use of health service” block were selected for this study, which concerns HPV vaccination.

### Sampling

The sample was sized by the National Institute of Educational Studies and Research Anísio Teixeira (INEP), which estimated population parameters representative of the population, composed of students from 13 to 17 years of age, from public and private schools in Brazil. The PeNSE sampling was performed by clusters in two stages, in which schools corresponded to the first stage of selection, and the classes of students enrolled corresponded to the second stage.

The selection of classes from each school was performed by simple random sampling. At this stage, the number of classes that should be selected was considered, according to the allocation stratum (geographic location and administrative dependence) to which the schools belonged. Thus, the sample of students comprised all of those from the classes selected from the schools that were selected in the first stage. All students present in class on the day of data collection were automatically selected to answer the survey questionnaire. Further methodological details about PeNSE sampling plan are available in another publication^12^.

The PeNSE sample was composed of 4,361 Brazilian public and private schools. Among the selected schools, 119 were not surveyed or could not have their information used, totaling 4,242 participating schools, representing 97.27% of the total schools predicted by the sample calculation.

Due to the complex sampling of the PeNSE, sampling weights were used, after data collection, in association with each student participating in the research who presented a questionnaire considered valid^12^. Moreover, the PeNSE 2019 database went through a process of evaluation and verification of the information, in order to standardize the data, adjust possible inconsistencies, and create derived variables necessary for the calculation of indicators^12^.

### Data source

PeNSE 2019 data collection was performed using the IBGE data collection network via structured and self-administered questionnaires made available to the participating adolescents on mobile collection devices. The data are in the public domain and are available on the IBGE website (https://www.ibge.gov.br/estatisticas/sociais/educacao/9134-pesquisa-nacional-de-saude-do-escolar.html?=&t=resultados).

### Participants

In this study, data from all schools and classes selected in the preparation stage of the research sampling plan were used. Participants were all students aged 13 to 17 years, enrolled from the 7^th^ grade of primary education to the 3^rd^ grade of secondary education, including technical courses with integrated high school and the regular/Teaching degree courses that were present on the day of data collection.

Schools with less than 20 students enrolled, classes with a low number of valid questionnaires in relation to the total number of enrolled students, and questionnaires that did not meet the eligibility criteria previously established by the data collection team^12^ were excluded from this study.

### Study variables

The variables of this study were the HPV vaccination status of adolescents (evaluated by the question: were you vaccinated against the HPV virus? Answer options: yes or no) and the reasons for non-vaccination (assessed by the question: why were you not vaccinated against the HPV virus? Answer options: did not know they had to get immunized; distance or difficulty to go to the unit or service; fear of reaction to the vaccine; did not believe in the effect of the vaccine; mother, father, or guardian did not want to vaccinate him; another reason). In the PeNSE 2019 database, questions were selected that investigated whether the adolescent was adequately vaccinated against HPV and, if not, what was the reason for non-vaccination. The prevalence of each response was estimated according to the categories of analysis (location, sex, and administrative dependence of the school).

### Bias control

Due to the complex sampling design of PeNSE and due to losses, post-stratification weights were considered for all analyses. Additionally, according to the IBGE publication, all research results went through a process of data evaluation and verification, in order to define which questionnaires would be considered as valid and, therefore, included in the research^12^.

### Data processing and analysis

**Figure 1:**
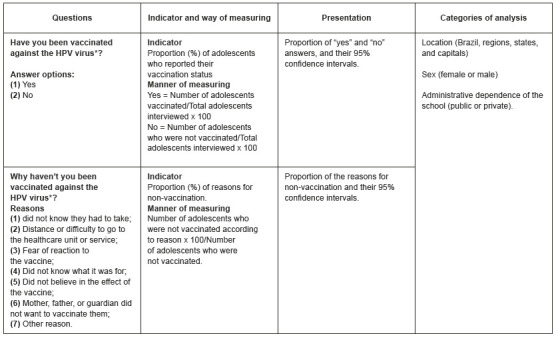
Indicators, questions, and answer options of the Questionnaire of the National Survey of School Health. Brazil, 2019 *HPV = Human papillomavirus

Initially, the prevalence of adolescents who answered on whether they were vaccinated against HPV was estimated, according to sex and administrative dependence of the school (public or private) with the respective 95% confidence interval (95%CI). Subsequently, the percentages of the reasons reported by the adolescents for not having vaccinated and the respective 95%CI were estimated, according to type of school administration (public or private) and location (region, federation units, and capitals) (Figure 1).

To evaluate the differences between the categories of analyses, estimates of 95%CI were considered, since the information returned by 95%CI is more valuable than the p-value^17^. Thus, the existence of statistically significant differences was considered when there was no overlap of 95%CI. Additionally, the Chi-square test was used to evaluate the differences between the prevalence of adolescents vaccinated against HPV according to sex, administrative dependence on the school, and regions of Brazil*.* Adjusted prevalence ratios (aPR) were also estimated for sex and 95%CI of the reasons for non-vaccination against HPV, according to the regions of Brazil and administrative dependence of the school, using the Poisson regression model with robust variance. To control possible confounding factors, the adjusted analysis considered the model proposed by Boakye et al.^18^. The adjustment was made since the literature reports that girls vaccinate more against HPV than boys^5^, making it possible to consider the influence of this sociodemographic factor^18^. To analyze the quality of the fit in the Poisson models, the adjustment quality test (F test) was used. A 5% significance level was adopted.

Data were collected using Microsoft Office Excel (Microsoft^©^, 2016) software, and statistical analyses were performed with Statistical Package for Social Sciences (SPSS), version 20.0, and Data Analysis and Statistical (Stata) software, version 14, using the survey module for complex samples, which incorporate post-stratification weights.

### Ethical aspects

PeNSE 2019 was approved through the opinion of the National Research Ethics Commission of the Ministry of Health (Conep/MS) no. 3,249,268, of April 8, 2019. All participants registered that they agreed to participate in the study through the Informed Consent Form (ICF). The participation was voluntary and ensured the confidentiality of the information obtained.

## Results

PeNSE 2019 estimated 11,851,941 schoolchildren aged 13 to 17 attending school in the country, 10,136,751 (85.5%) enrolled in public schools and 1,715,190 (14.5%) in private schools. The sample of this study consisted of 159,245 schoolchildren aged 13 to 17 years, corresponding to 84.72% of the total initially predicted to be part of the study sample, 840 (84.06%) living in the North, 1,703 (84.78%) in the Northeast region, 730 (86.10%) in the Southeast region, 460 (85.65%) in the South, and 628 (86.24%) in the Midwest region.

**Figure 2:**
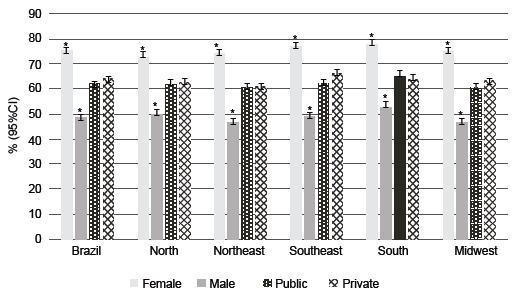
Prevalence of schoolchildren aged 13 to 17 years who were vaccinated against human papillomavirus, according to sex, administrative dependence of the school, with indication of 95% confidence intervals, according to regions. National Survey of School Health, Brazil, 2019. 95%CI = 95% confidence interval. Note: No results are presented for students who left unanswered. *p-value<0.05 = indicates statistically significant differences by chi-square test

In Brazil, 62.9% (95%CI 62.1;63.6) of the students who participated in PeNSE reported that they were vaccinated against HPV. Regarding sex, the proportion of immunized girls was higher than that of boys, corresponding to 76.1% (95%CI 75.3;77.0) and 49.1% (95%CI 48.2;50.1), respectively (Figure 2).

Regarding the administrative dependence of the school and the regions, there was a difference in the prevalence of those vaccinated between females and males. However, there was no difference in the prevalence of those vaccinated among students from public and private schools in all regions of Brazil (Figure 2).

**Figure 3:**
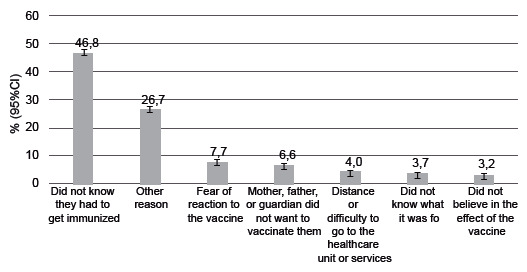
Percentage of the reasons reported for not vaccinating against human papillomavirus among Brazilian schoolchildren between 13 and 17 years old, with indication of 95% confidence intervals. National Survey of School Health, Brazil, 2019 95%CI = 95% Confidence Interval.

As for the reasons for not receiving the vaccine, most adolescent students in the country answered “did not know they had to take” (46.8%; 95%CI 45.4;48.3), followed by the answers “other reason” (26.7%; 95%CI 25.4;27.9) and “fear of reaction to the vaccine” (7.7%; 95%CI 6.9;8.6), respectively (Figure 3).

We noticed that aPR were higher among Brazilian schoolchildren from public schools for reasons related to “Did not know they had to get immunized” (1.6; 95%CI 1.5;1.7) and “Distance or difficulty to go to the unit or service” (1.7%; 95%CI 1.3;2.2); for private school children, the “fear of reaction to the vaccine” (1.4; 95%CI 1.1;1.6), “mother, father, or guardian did not want to vaccinate them” (2.5; 95%CI 2.2;3.0), and “other reasons” (1.4; 95% CI 1.3;1.5) (Table 1).

According to the regions, we observed that the aPR were higher for the following reasons: “Did not know they had to get immunized” in adolescents from public schools (1.2; 95%CI 1.1;1.2) and private (1.1; 95%CI 1.1;1.2) from the Northeast region, and in private school children (1.1; 95%CI 1.1;1.2) from the North region; “distance or difficulty to go to the unit or service” in adolescents from public schools (2,3; 95%CI 1.6;3.2) and private (1.6; 95%CI 1.1;2.3) from the North region; “fear of reaction to the vaccine” in adolescents from private schools in the Northeast region (1.6; 95%CI 1.1;2.3); “did not know what it was for” in public school children in the Midwest (1.4; 95%CI 1.1;1.8); “did not believe in the effect of vaccines” in schoolchildren from private schools in the South region (2.2; 95%CI 1.6;3.1); “mother, father, or guardian did not want to vaccinate them” in adolescents from public schools in the Northeast regions (1.7; 95%CI 1.3;2.3) and Midwest (1.3; 95%CI 1.1;1.8) and in private schools in the South region (1.3; 95%CI 1.1;1.5) (Table 1).

**Table 1:** Percentage and prevalence ratios adjusted for the reasons for non-vaccination against human papillomavirus among Brazilian schoolchildren aged 13 to 17 years, according to administrative dependence of the school and regions, with indication of 95% confidence intervals. National Survey of School Health, Brazil, 2019

Administrative dependency and large regions		Indicators													
		Did know they had to get immunized		Distance or difficulty to go to the unit or service		Fear of vaccine reaction		Did not know what it was for		Did not believe in the effect of the vaccine		Mother, father, or guardian did not want to vaccinate them		Other reason	
		% (95%CI)	aPR (95%CI)	% (95%CI)	aPR (95%CI)	% (95%CI)	aPR (95%CI)	% (95%CI)	aPR (95%CI)	% (95%CI)	aPR (95%CI)	% (95%CI)	aPR (95%CI)	% (95%CI)	aPR (95%CI)
Public	Brazil	49.2 (47.6;50.9)	1.6 (1.5;1.7)*	4.2 (3.4;4.9)	1.7 (1.3;2.2)*	7.3 (6.4;8.3)	0.7 (06;0.9)*	3.8 (3.1;4.4)	1.2 (0.9;1.5)	3.2 (2.5;38)	0.8 (0.7;1.1)	5.5 (4.6;6.4)	0.4 (0.3;0.4)*	25.4 (24;26.8)	0.7 (0.7;0.8)*
	North	46.3 (41.7;50.9)	1.0 (0.9;1.0)	7.9 (4.6;11.2)	2.3 (1.6;3.2)*	5.7 (4.0;7.4)	0.8 (0.6;1.0)	2.8 (1.6;4.1)	0.7 (0.5;1.0)	2.8 (1.5;4.1)	0.9 (0.5;1.4)	4.6 (3.0;6.3)	0.8 (0.5;1.2)	27.9 (24.6;31.2)	1.1 (0.9;1.2)
	Northeast	54.6 (52.0;57.2)	1.2 (1.1;1.2)*	4.5 (3.5;5.6)	1.2 (0.9;1.5)	6.8 (5.4;8.3)	0.9 (0.7;1.1)	3.3 (2.5;4.1)	0.9 (0.7;1.3)	2.4 (1.6;3.2)	0.7 (0.5;1.1)	3.1 (2.3;3.9)	0.4 (0.3;0.6)*	24.1 (21.9;26.2)	0.9 (0.8;1.0)
	Southeast	47.2 (43.8;50.6)	0.9 (0.9;1.0)	3.1 (1.8;4.4)	0.7 (0.4;0.9)*	7.8 (5.8;9.9)	1.1 (0.9;1.4)	3.4 (2.0 ;4.8)	0.9 (0.6;1.4)	3.9 (2.4;5.4)	1.3 (0.9;2.0)	7.8 (5.4;10.3)	1.7 (1.3;2.3)*	25.2 (22.2;28.1)	1.0 (0.9;1.2)
	South	46.3 (42.2;50.4)	0.9 (0.8;1.0)	1.6 (0.5;2.7)	0.3 (0.2;0.6)*	9.7 (6.9;12.5)	1.2 (0.9;1.7)	5.8 (3.5;8.2)	1.3 (0.9;2.0)	3.5 (2.2;4.8)	1.0 (0.7;1.6)	5.5 (3.8;7.3)	1.4 (1.0;1.8)	26.9 (23.2;30.7)	1.1 (1.0;1.3)
	Midwest	45.5 (42.2;48.9)	0.9 (0.8;0.9)*	4.7 (3.3;6.1)	1.0 (0.8;1.4)	7.0 (5.1;8.9)	1.0 (0.8;1.3)	5.7 (4.2;7.1)	1.4 (1.1;1.8)*	3.5 (2.4;4.7)	1.2 (0.8;1.7)	6.7 (4.7;8.8)	1.3 (1.1;1.8)*	25.3 (22.6;27.9)	1.0 (0.9;1.1)
Private	Brazil	31.4 (29.6;33.2)	0.6 (0.6;0.7)*	2.6 (2.0;3.1)	0.6 (0.5;0.7)*	10.2 (8.8;11.6)	1.4 (1.1;1.6)*	3.1 (2.5;3.7)	0.9 (0.7;1.1)	3.4 (2.8;4.1)	1.2 (0.9;1.5)	14.0 (12.5;15.5)	2.5 (2.2;3.0)*	34.8 (33.1;36.5)	1.4 (1.3;1.5)*
	North	40.9 (36.5;45.3)	1.3 (1.2;1.4)*	3.2 (1.9;4.5)	1.2 (0.8;2.0)	6.1 (4.4;7.9)	0.6 (0.4;0.8)*	2.9 (1.6;4.2)	0.8 (0.5;1.2)	2.7 (1.0;4.3)	0.8 (0.5;1.5)	11.2 (8.6;13.7)	0.8 (0.7;1.0)	32.0 (28.9;35.2)	0.9 (0.8;1.1)
	Northeast	34.9 (32.8;37.0)	1.1 (1.1;1.2)*	3.2 (2.4;4)	1.6 (1.1;2.3)*	7.8 (6.7;8.9)	1.6 (1.1;2.3)*	3.0 (2.3;3.8)	0.9 (0.7;1.2)	2.1 (1.5;2.7)	0.6 (0.4;0.8)*	12.2 (10.8;13.7)	0.8 (0.7;0.9)*	36.4 (34.3;38.4)	1.0 (1.0;1.3)
	Southeast	29.6 (25.9;33.4)	0.9 (0.8;1.0)	2.7 (1.6;3.8)	0.9 (0.5;1.3)	13.1 (10.0;16.2)	1.6 (1.2;2.0)	3.1 (2;4.2)	1.2 (0.8;1.7)	3.1 (2.0;4.2)	0.9 (0.6;1.3)	14.1 (10.9;17.3)	1.0 (0.9;1.2)	33.8 (30.4;37.2)	0.9 (0.8;1.1)
	South	24.5 (21.2;27.8)	0.8 (0.7;0.9)*	0.9 (0.2;1.5)	0.3 (0.2;0.6)*	9.2 (7.0;11.5)	0.8 (0.6;1.0)	3.1 (1.8;4.4)	0.9 (0.6;1.4)	7.2 (4.9;9.5)	2.2 (1.6;3.1)*	17.8 (14.7;20.8)	1.3 (1.1;1.5)*	36.9 (33.4;40.4)	1.1 (1.0;1.2)
	Midwest	32.5 (29.5;35.5)	1.0 (0.9;1.0)	2.3 (1.4;3.2)	1.0 (0.7;1.4)	7.9 (6.3;9.5)	0.8 (0.6;0.9)*	3.4 (2.3;4.6)	1.0 (0.7;1.4)	4.4 (3.0;5.8)	1.3 (0.8;1.7)	15.3 (12.8;17.8)	1.1 (0.9;1.3)	33.8 (30.8;36.7)	1.0 (0.9;1.1)

Note: No results are presented for students who left unanswered.

aPR= Prevalence ratio adjusted for sex. 95%CI: 95% confidence interval.

*Corresponds to the p-value of the aPR < 0.05, indicating statistically significant differences calculated by the Poisson regression model.

As for the administrative dependence of the school, in addition to the answer “Did not know that they had to get immunized” (public school: 49.2%; 95%CI 47.6;50.9; private school: 31.4%; 95%CI 29.6;33.2), the answers “distance or difficulty to go to the unit or service” (4.2%; 95%CI 3.4;4.9) and “fear of reaction to the vaccine” (7.3%; 95%CI 6.4;8.3) were more frequent in adolescent schoolchildren from public schools, while the answer “mother, father, or guardian did not want to vaccinate them” (14%; 95%CI 12.5;15.5) was more frequent in adolescents from private schools (Table 1).

Regarding the reasons, the answer “Did not know they had to get immunized” was more frequent in the states of the North and Northeast regions: Alagoas (62.9%; 95%IC 57.3;68.6), Maranhão (52.8%; 95%CI 46.0;59.5), Piauí (60.4%; 95%CI 54.8;66.1), Pernambuco (55.6%; 95%CI 49.3;62.0), and Sergipe (52.1%; 95%CI 46.9;57.4) (Table 2). We noticed that there were differences in the distribution of the reasons for non-vaccination against HPV in schoolchildren according to federative units and regions (p < 0.05) (Table 2).

**Table 2:** Percentage of the reasons for non-vaccination against human papillomavirus in schoolchildren aged between 13 and 17 years, according to the regions and units of the federation, with indication of 95% confidence intervals. National Survey of School Health, Brazil, 2019

Regions and federative units^*^		Indicators						
		Did not know they had to take	Distance or difficulty to go to the unit or service	Fear of vaccine reaction	Did not know what it was for	Did not believe in the effect of the vaccine	Mother, father, or guardian did not want to vaccinate them	Other reason
		% (95%CI)	% (95%CI)	% (95%CI)	% (95%CI)	% (95%CI)	% (95%CI)	% (95%CI)
**North**		46.0 (41.7;50.3)	7.6 (4.5;10.7)	5.7 (4.2;7.3)	2.8 (1.6;4.0)	2.8 (1.6;4.0)	5.0 (3.5;6.6)	28.1 (25.1;31.2)
	Rondônia	49.7 (45.1;54.2)	3.1 (1.6;4.6)	8.4 (5.6;11.3)	3.6 (1.6;5.6)	1.0 (0.3;1.7)	5.0 (3.3;6.8)	27.4 (23.6;31.2)
	Acre	44.2 (39.8;48.5)	2.6 (1.1;4.1)	11.4 (8.9;13.9)	3.7 (2.0;5.4)	2.1 (0.7;3.5)	7.1 (4.8;9.4)	26.2 (22.4;30.0)
	Amazonas	49.5 (43.1;56)	5.7 (2.3;9.1)	4.8 (2.6;7)	3.7 (1.3;6.0)	3.0 (1.0;5.0)	4.2 (2.0;6.4)	29.1 (23.5;34.6)
	Roraima	49.4 (44.0;54.7)	5.6 (3.2;7.9)	5.9 (3.5;8.3)	4.9 (2.4;7.3)	3.2 (0.4;5.9)	5.9 (3.7;8.2)	23.7 (18.8;28.6)
	Pará	43.3 (35.9;50.7)	10.2 (4.9;15.6)	4.8 (2.0;7.7)	2.0 (0.0;3.9)	3.5 (1.3;5.6)	5.0 (2.4;7.7)	29.1 (23.7;34.6)
	Amapá	50.2 (45.2;55.1)	3.6 (2.0;5.2)	6.3 (4.1;8.5)	4.8 (3.1;6.4)	1.8 (0.8;2.8)	8.2 (5.6;10.8)	23.0 (19.4;26.6)
	Tocantins	50.7 (42.6;58.8)	4.7 (2.0;7.4)	6.7 (4.2;9.2)	3.5 (1.9;5.0)	0.6 (^†^;1.4)	3.0 (1.5;4.5)	26.3 (20.6;32)
**Northeast**		52.2 (49.9;54.5)	4.4 (3.5;5.3)	6.9 (5.6;8.2)	3.3 (2.6;4.0)	2.3 (1.6;3.1)	4.2 (3.5;4.9)	25.5 (23.6;27.5)
	Maranhão	52.8 (46;59.5)	7.3 (4.4;10.3)	5.7 (3.6;7.8)	3.8 (1.6;6.0)	2.1 (0.3;3.9)	1.9 (0.9;2.8)	24.0 (19.8;28.3)
	Piauí	60.4 (54.8;66.1)	2.8 (1.1;4.6)	4.0 (2.0;6.1)	2.6 (1.2;4.0)	2.9 (1.3;4.4)	2.5 (1.3;3.7)	23.6 (19.1;28.1)
	Ceará	49.4 (43.4;55.5)	4.2 (1.3;7)	7.4 (3.6;11.3)	2.9 (1.4;4.4)	3.4 (0.4;6.5)	2.9 (1.7;4.2)	29.1 (24.4;33.7)
	Rio Grande do Norte	48.3(44.4;52.2)	2.7 (1.4;4)	8.7 (6.1;11.3)	4.4 (2.4;6.4)	3.1 (1.7;4.5)	5.2 (3.8;6.6)	26.5 (23.0;30.0)
	Paraíba	53.3 (49.3;57.2)	4.3 (2.3;6.2)	5.3 (3.6;7.0)	2.2 (1.1;3.3)	2.0 (0.9;3.1)	6.7 (4.5;8.9)	24.9 (21.1;28.6)
	Pernambuco	55.6 (49.3;62)	3.9 (1.9;5.8)	5.5 (2.7;8.2)	2.8 (1.3;4.4)	2.3 (0.9;3.7)	5.3 (2.9;7.7)	24.0 (18.9;29.1)
	Alagoas	62.9 (57.3;68.6)	3.7 (1.3;6.0)	6.1 (3.5;8.7)	3.3 (1.4;5.2)	1.5 (^†^;3.1)	2.9 (1.3;4.4)	18.9 (13.0;24.8)
	Sergipe	52.1 (46.9;57.4)	2.2 (1.0;3.4)	5.6 (3.6;7.5)	4.2 (2.1;6.4)	2.5 (0.9;4)	5.7 (4.1;7.3)	26.9 (23.4;30.5)
	Bahia	47.0 (41.3;52.7)	4.2 (2.0;6.4)	10.0 (5.6;14.4)	3.5 (1.5;5.4)	1.7 (0.2;3.3)	5.6 (3.5;7.8)	27.2 (21.1;33.3)
**Southeast**		44.2 (41.3;47.1)	3.0 (1.9;4.1)	8.7 (6.9;10.5)	3.4 (2.2;4.5)	3.7 (2.5;5)	8.9 (6.8;11.0)	26.6 (24.1;29.2)
	Minas Gerais	47.1(38.5;55.8)	3.2 (0.6;5.8)	3.3 (1.4;5.3)	5.9 (2.2;9.7)	3.5 (1.1;5.9)	5.6 (3.0;8.3)	28.2 (20.6;35.8)
	Espírito Santo	40.0 (32.8;47.3)	2.2 (0.4;3.9)	6.8 (3.4;10.2)	6.4 (2.7;10.1)	4.3 (1.1;7.4)	11.3 (6.6;15.9)	28.1 (21.7;34.4)
	Rio de Janeiro	36.9 (31.9;41.8)	4.3 (2.3;6.4)	12.7 (8.5;16.9)	3.8 (2.3;5.3)	3.2 (1.6;4.8)	9.4 (6.2;12.6)	28.1 (24.2;31.9)
	São Paulo	46.9 (43.0;50.7)	2.4 (0.9;3.9)	8.8 (6.3;11.4)	2.1 (0.5;3.6)	4.1 (2.0;6.2)	9.7 (6.1;13.2)	25.3 (21.8;28.8)
**South**		43.0 (39.4;46.6)	1.5 (0.5;2.4)	9.6 (7.2;12.0)	5.4 (3.5;7.4)	4.0 (2.9;5.2)	7.4 (5.8;9.0)	28.4 (25.2;31.6)
	Paraná	41.7 (34.6;48.8)	1.8 ^(†^;3.6)	8.5 (4.3;12.8)	7.8 (2.8;12.8)	3.6 (1.7;5.6)	6.2 (3.5;8.9)	29.2 (22.5;36.0)
	Santa Catarina	35.3 (29.4;41.3)	1.2 (^†^;2.3)	14.6 (8.7;20.5)	4.1 (1.7;6.4)	5.4 (3.0;7.8)	7.4 (4.6;10.3)	31.3 (25.5;37.0)
	Rio Grande do Sul	48.6 (43.3;53.9)	1.5 (^†^;3.1)	7.4 (4.4;10.3)	4.7 (2.0;7.4)	3.5 (1.7;5.2)	8.1 (5.5;10.8)	26.2 (21.5;30.8)
**Midwest**		43.7 (40.8;46.7)	4.4 (3.2;5.6)	7.1 (5.5;8.8)	5.4 (4.1;6.6)	3.7 (2.6;4.7)	7.9 (6.1;9.7)	26.5 (24.1;28.8)
	Mato Grosso do Sul	44.9 (40.4;49.5)	2.6 (1.3;3.9)	8.5 (4.7;12.3)	7.9 (5.3;10.6)	5.9 (2.8;9.0)	5.8 (3.5;8.1)	24.3 (18.4;30.2)
	Mato Grosso	47.8 (41;54.6)	3.2 (1.2;5.2)	8.5 (3.9;13.1)	4.2 (0.7;7.6)	1.7 (0.2;3.2)	8.8 (4.0;13.6)	22.8 (17.3;28.4)
	Goiás	42.1 (37.7;46.4)	4.9 (2.8;7.0)	6.9 (4.8;9.0)	6.0 (4.1;7.9)	3.6 (2.0;5.3)	6.4 (4.5;8.4)	29.2 (25.8;32.6)
	Distrito Federal	40.7 (32.5;48.9)	6.6 (2.7;10.6)	4.4 (2.2;6.7)	3.0 (1.1;5.0)	4.3 (2.0;6.6)	12.4 (6.6;18.1)	27.0 (22.6;31.5)

Note: No results are presented for students who left unanswered.

*Corresponds to the p-value < 0.05, indicating statistically significant differences by the Chi-square *test*; ^†^Corresponds to intervals where it was not possible to estimate the lower limit of the confidence interval

Regarding the prevalence of schoolchildren who were not vaccinated against HPV in the State capitals, Rio Branco, Natal, Porto Alegre, and Macapá reached the highest percentages, corresponding to 22.1% (95%CI 19.6;24.6), 21.3% (95%CI 18.9;23.7), 20.4% (95%CI 17.7;23.0), 18.8% (95%CI 16.2;21.3), respectively.

The capitals of the Northern and Northeastern States presented higher proportions of the response “did not know they had to take”; most notably, in Teresina (54.7%; 95%CI 50.5;58.8), Maceió (54.6%; 95%CI 49.4;59.9), and Boa Vista (51%; 95%IC 44.5;57.5). In turn, the capitals of Florianópolis 30.9% (95%CI 24.7;37.2), Porto Alegre 33.6% (95%CI 27.5;39.8), and Vitória 36.6% (95%CI 30.7;42.5), located in the South and Southeast regions, had the lowest prevalence of this response.

## Discussion

Most of the schoolchildren aged 13 to 17 years who participated in PeNSE were vaccinated against HPV, with a higher prevalence of vaccination among girls than boys. In Brazil and in public schools of the Northeast region, the most frequent reason for non-vaccination was “Did not know they had to take it.” In addition to this reason, the “distance or difficulty to go to the unit or service” was more frequent among adolescents enrolled in Brazilian public schools. Schoolchildren enrolled in private schools responded more frequently that the “mother, father, or guardian did not want to vaccinate him” and that they were “afraid of having a reaction to the vaccine.” A higher prevalence of adolescents who were not vaccinated against HPV was observed in the capitals and States of the North and Northeast regions.

Notably, when the HPV vaccine was included in the vaccination schedule of adolescents in 2014, the vaccination strategy in schools favored reaching the goal of 80% of vaccination coverage in just three months^19^. However, the outbreak of psychogenic reaction associated with HPV vaccine in girls from a school in Bertioga, São Paulo Brazil, which had wide media dissemination, was possibly the main event responsible for reducing the acceptance of HPV vaccine by the adolescent public^19^.

A systematic review that adopted interviews directed to this public as a methodological resource indicated that there are few studies that investigate the attitudes, perceptions, and practices of adolescents regarding HPV vaccination^11^. We also emphasize on the importance of these studies in educating those responsible for adolescents about behaviors and attitudes that configure risk factors for the development of sexually transmitted infections, and that affects the sexual and reproductive health of their children^20^ ^), (^ ^21^. A study conducted in Mexico investigated the reasons for non-vaccination against HPV^22^. In Brazil, we did not find studies in which population surveys were used to investigate the reasons why adolescents are not adhering to HPV vaccination, reinforcing the importance and the need for further studies that allow further discussion on this topic.

The unawareness of HPV vaccination was the most frequent reason among those listed to justify non-vaccination by schoolchildren. This result was also reported by a study with data from PeNSE 2015, revealing that 10.30% of the students did not know or had not heard of the HPV vaccination campaign^5^. The same study found a positive association between the unawareness of the campaign against HPV and the age group from 15 to 17 years, studying in the afternoon/night shift, having had sex, presenting self-perception of health status as poor or very poor, being dissatisfied or indifferent about body image, and studying in public school^5^. In addition to the unawareness about the campaigns, international studies indicate that the lack of adequate information about HPV and religious beliefs stood out as reasons for non-vaccination^11^ ^), (^ ^21^ ^), (^ ^23^. Many parents considered that by accepting the vaccination of their daughters, they would be legitimizing sexual behaviors inappropriate for their age^23^.

Despite the importance of disseminating information on HPV to increase vaccination adherence by the adolescent public^5^ ^), (^ ^24^ ^), (^ ^25^, evidence shows that many health professionals do not discuss or recommend this protection method^14^. Moreover, inadequate communication strategies are adopted, compromising the acceptance of the vaccine by adolescents^14^. In this sense, nurses should make efforts to establish fruitful and favorable communication channels when sharing information with adolescents and their families about HPV and the importance of vaccination.

In turn, having knowledge about the virus and about the action of the vaccine was a factor positively associated with vaccination of the adolescent public within national and international studies^5^ ^), (^ ^25^. Regarding the source of information about HPV, a multicenter study with sexually active young adults recruited from 119 Primary Health Care (PHC) services revealed higher scores on knowledge about the virus among participants who were informed via health professionals and the media^23^. The research reinforced the importance of nurses working in these services of adopting communication strategies that favor the dissemination of information, favoring the adherence of this public to vaccination^21^ ^), (^ ^26^.

Health professionals, in addition to providing information on HPV vaccination, should recommend it to adolescents^14^ ^), (^ ^21^ ^), (^ ^22^ ^), (^ ^23^ ^), (^ ^24^ ^), (^ ^25^ ^), (^ ^26^. Studies show that parents who received the recommendation from a health professional have a higher chance of reporting their intention to vaccinate their children when compared to parents who did not^14^ ^), (^ ^26^. Another study, however, revealed that only 64.4% of the parents of girls and 41.6% of the parents of boys received recommendations from health professional regarding the vaccine^27^. Notably, a study conducted with nurses and other health professionals in Nigeria reported that knowledge about the HPV vaccine is favored by its recommendation to parents and adolescents^26^. Considering that in our study most adolescents did not know that they had to be vaccinated and that there is evidence that knowledge about the vaccine is favored by its recommendation from a health professional^26^, strategies that prioritize the training of nurses on HPV prevention are necessary^26^, especially those who work in the vaccine rooms.

Regarding the administrative dependence of the school, there was a higher prevalence of adolescents enrolled in public schools who answered “did not know they had to take it.” Another study, which analyzed data from the third edition of PeNSE 2015, also identified that in public schools there is a higher prevalence of unawareness about HPV vaccination^5^. Notably, the School Health Program (PSE), established in 2007 by Decree No. 6,286 of December 5, 2007, is part of the intersectoral health and education policy and aims to improve the health of schoolchildren enrolled in public schools in Brazil^28^. However, the adoption of inadequate methodologies to address the prevention of sexually transmitted infections and sexual and reproductive rights can hinder the awareness of adolescents regarding the importance of HPV vaccination as prevention^28^.

Considering that an analysis by sex is essential for the proper evaluation of health indicators in Brazil and that the results of our study showed a lower proportion of male adolescents vaccinated against HPV compared to females in all regions of the country, interventions should consider gender differences when developing health strategies aimed at improving immunization indicators in this group. A study with data from PeNSE 2015 also showed a higher proportion of unvaccinated male adolescents and a positive association between males and unawareness of the HPV campaign^5^.

In addition to preventing cervical cancer, the HPV vaccine also prevents penile cancer and other types that affect individuals of both sexes^24^, which reinforces the importance of educating male adolescents and young adults on HPV vaccination. A study with PNI data also drew attention to the differences in HPV vaccine coverage in the female and male population^25^. In the period from 2013 to 2018, 317 municipalities (5.7%) reached the goal of at least 80% of the female population aged 9 to 13 years vaccinated with both doses of the HPV vaccine and only 23 municipalities (0.4%) reached the goal of at least 80% of the male population aged 11 to 14 years adequately immunized^19^.

The inadequate approach to sexual and reproductive rights associated with socially instituted differences contributes to the naturalization of the responsibility of female adolescents for the prevention of pregnancy, as well as for the prevention of sexually transmitted diseases, including HPV^29^. In this sense, in addition to knowing the factors that compromise the achievement of vaccine coverage goals, it is essential that nurses working in PHC services develop health strategies that reach adolescents and their families, adopting culturally appropriate, flexible methodologies that educate them on the importance of HPV vaccination^14^. The bond of nurses with families and the recommendation of vaccination by these professionals increases the adherence of adolescents and their guardians to the campaigns, according to a study conducted on the African continent^26^.

In our study, the North and Northeast regions had the highest prevalence of adolescents not vaccinated against human papillomavirus in the country. During the COVID-19 pandemic, the North and Northeast regions of Brazil also showed a reduction in the number of doses applied for HPV vaccine, contributing to the increase in the number of adolescents of both sexes who did not have access to primary prevention of cervical cancer in these regions^30^. In this sense, health strategies and policies are necessary to increase the support of the adolescent public living in these regions, especially in the Northeast, which has, in addition to the worst indicators of immunization against HPV^19^, the higher prevalence of cervical cancer and other neoplasms caused by the virus^24^.

In Brazil, regional inequalities in vaccination coverage are historical and significant, and the worst indicators of immunization are commonly identified within the States and municipalities of the North and Northeast regions, when compared to the South and Southeast regions^31^ ^), (^ ^32^. A national study that estimated the coverage of the first and second dose of the HPV vaccine in cohorts of girls aged 14, 15, and 16 years in 2017^9^ also identified heterogeneity of vaccination coverage, in addition to an association between the proportion of households without private bathrooms in the municipality and the worst indicators of immunization^9^. Also in this study, the States of Amazonas, Pará, Tocantins, Piauí, Paraíba, and Bahia, located in the North and Northeast regions, did not reach the goal of 80% of the vaccination coverage of the target population^9^.

We emphasize the importance of continuing the health monitoring of Brazilian adolescents through PeNSE, since this survey allows the identification of risk and protective factors for the health of adolescents, and this information is fundamental for the development of policies aimed at improving the health indicators of this population^12^. The data collection instrument of the fourth edition of PeNSE was reviewed and updated, resulting in the modification and exclusion of some questions and the inclusion of others. These alterations favor the adolescent’s understanding of the instrument^12^, but hinder the comparison of some indicators. In the fourth edition of PeNSE 2019, for example, questions that investigated the reason for non-vaccination were included, which represents an advance, and we recommend for this question to be maintained in future editions of this survey. Thus, this scenario favors the comparison with future editions, since the 2019 edition had its data collected before the COVID-19 pandemic, allowing for it to be a baseline for evaluations in a post-pandemic setting.

In this study, memory bias represented a limitation since adolescents had to evoke previous facts to answer questions from the PeNSE questionnaire. Additionally, the target audience of the research tends to answer complex questions inaccurately, which may lead to underestimation or overestimation of the information provided^5^. The fear of judgment and feelings of shame from prior experience may prevent students from responding in a reliable way, representing a potential risk of obtaining inadequate information^25^. Considering the negative impacts of non-vaccination against HPV on the health of adolescents, this study advances by identifying, in an unprecedented way, the population prevalence of vaccination against the disease among schoolchildren in Brazil via the PeNSE 2019 data. The last PeNSE survey that collected information on HPV vaccination in this population was conducted in 2015^33^. In this sense, knowing the current panorama of HPV vaccination with the available data is extremely relevant for the monitoring of this indicator in the country.

This research also advanced by bringing information on HPV vaccination for both sexes, since in the 2015 edition of PeNSE HPV vaccination was investigated exclusively in female adolescents. This is the first study that investigated the reasons for non-vaccination against HPV, using the PeNSE 2019 database. Therefore, the results of this research may support public policies and health strategies for the control and prevention of cervical cancer in the country.

Regarding other possibilities, we highlight the importance of the role of nurses in the development of health strategies in interinstitutional spaces, adopting culturally appropriate, flexible methodologies that consider the singularities of the adolescent public. Among these spaces, the school environment deserves attention for providing opportunities for the encounter and establishment of a communication channel favorable to clarifying doubts and addressing aspects potentially associated with HPV vaccine adherence, such as fear of adverse effects after vaccination and prevention of cervical cancer^5^ ^), (^ ^24^ ^), (^ ^25^.

## Conclusion

The strengthening of public policies and health strategies, especially in the North and Northeast regions of the country, is essential to improve HPV vaccination indicators among the adolescent public. It is also worth mentioning the central role of nurses as a health educator, establishing a communication channel that provides information on vaccination against the virus, which can contribute to increased vaccination adherence among Brazilian adolescents.
